# Modelling the mass adoption of mobile payment for e-hailing services using SEM-MGA

**DOI:** 10.1371/journal.pone.0287300

**Published:** 2023-10-13

**Authors:** Siyu Long, Abdullah Al Mamun, Qing Yang, Jingzu Gao, Wan Mohd Hirwani Wan Hussain, Sayed Samer Ali Al Shami

**Affiliations:** 1 UKM—Graduate School of Business, Universiti Kebangsaan Malaysia, UKM Bangi, Selangor Darul Ehsan, Malaysia; 2 Institute of Technology Management and Entrepreneurship, Universiti Teknikal Malaysia Melaka, Melaka, Malaysia; National University of Sciences and Technology, PAKISTAN

## Abstract

Secured financial transactions can now be conveniently made via mobile devices for various products and services, such as e-hailing. However, limited research exists on the factors influencing the adoption of mobile payments specifically for e-hailing services, despite the growing interest in mobile payments in China. This cross-sectional study quantitatively assessed the influence of perceived usefulness, perceived ease of use, social influence, facilitating conditions, perceived security, and lifestyle compatibility on the intention to adopt and the actual adoption of mobile payment for e-hailing services. An online self-administered survey was conducted, involving 413 respondents from China. The results revealed a significant positive influence of perceived ease of use, social influence, facilitating conditions, and perceived security on the intention to adopt mobile payment. Additionally, the study found that the intention to adopt mobile payment positively influenced the actual adoption of mobile payments. Meanwhile, perceived usefulness and lifestyle compatibility demonstrated an insignificant influence on the intention to adopt mobile payments. Subgroup analysis further revealed gender-based differences, indicating that the influence of the intention to adopt mobile payment on the adoption of mobile payment for e-hailing services varied significantly between male and female respondents. Furthermore, the influence of facilitating conditions on the intention to adopt mobile payment for e-hailing services also differed significantly among respondents of different age groups. These findings contribute to a better understanding of the factors influencing the adoption of mobile payment for e-hailing services and provide insights for service providers and policymakers in promoting its adoption.

## Introduction

The expansion of the mobile Internet and the rise of mobile services have contributed to the increasing reliance on smart technologies and the growing popularity of mobile payment (m-payment) among consumers [1–5]. Unlike conventional payment methods, m-payment enables consumers to make financial transactions from anywhere at any time using their mobile devices [3]. As consumers increasingly rely on mobile services for their daily needs, including e-hailing services [6], the use of mobile applications for e-hailing services has grown owing to the popularity of smartphones and the need for convenience in daily travel [7]. The COVID-19 pandemic has further accelerated the adoption of m-payments as the preferred payment method for e-hailing services [8].

While mobile payments have gained popularity globally, the market in China is dominated by two major third-party mobile payment companies: Alipay and WeChat Pay [9]. These companies have revolutionized payment methods in China, with mobile payments accounting for the majority of transactions [8]. Additionally, the emergence and diffusion of e-hailing services has stimulated the development of the transportation service industry in China and the establishment of companies that provide e-hailing services. Products and services related to e-hailing in China have undergone a period of exploration and development, rapid expansion, and adjustment in the last decade and have gradually become a necessity in the daily lives of the Chinese masses [10]. The large user base, abundant labor force, favorable online innovation environment, and continuously increasing consumer demand in the Chinese market have enabled China’s e-hailing market to grow rapidly and even compensate for the shortcomings of the existing public transportation system [10, 11]. According to the Statistical Report on the Development of the Internet in China [12], there are approximately 350 million registered users of major e-hailing service software and applications in most cities in China. Thus, e-hailing is a major market segment in China. With the rapid growth in m-payments and their convenience, more e-hailing users opt to make contactless payments [3, 7]

M-payments have been explored in various contexts in various countries, including China [2, 3, 5] E-hailing applications have been promoted and popularized in China for nearly ten years, and their convenience has become a part of Chinese consumers’ daily travel plans. The e-hailing market in China has brought about transformative changes in urban transportation, making city travel more convenient and affordable while reducing traffic congestion and improving air quality [7]. In addition, e-hailing services have had a significant impact on employment, with many people in China now working as full-time or part-time drivers for companies such as Drip and Meituan [3]. However, there is a paucity of research on Chinese users’ use of mobile payment services specifically in the context of e-hailing. Existing studies have primarily focused on user groups, market dynamics, and policy considerations related to e-hailing services, overlooking user perceptions and acceptance of mobile payments for e-hailing [10, 13–15]. Prior studies on e-hailing services in China have mainly focused on the perspectives of e-hailing drivers, the transportation industry, and the taxi industry, but have overlooked the viewpoints of e-hailing users [11, 13, 15, 16]. Acknowledging the significance of m-payment as an alternative to conventional payment methods, the current study expected significant market expansion for m-payment and e-hailing services in China, given the close relationship between them. It was deemed noteworthy for the current study to examine factors that influence the intention to adopt m-payment (IN) and adoption of m-payment (AD) for e-hailing services among Chinese consumers.

Despite the popularity of e-hailing services and mobile payments, there was limited research on the use of mobile payment services by Chinese users in the context of e-hailing [7, 8, 11, 15]. Previous studies primarily focused on user groups, market dynamics, and policy considerations related to e-hailing services, while overlooking user awareness and acceptance of mobile payments for e-hailing [6, 7]. The majority of prior studies on e-hailing services adopted the theory of planned behavior (TPB) and the technology acceptance model (TAM) [6, 7]. This study aimed to address the existing research gaps regarding the demand, acceptance, and perception of e-hailing services and mobile payments in the Chinese market. Specifically, the study sought to understand the factors that influenced Chinese users’ intentions to adopt and the actual adoption of mobile payments for e-hailing services. To achieve this, the study employed the TAM as a framework to assess the influence of potential predictors (IN and AD) among e-hailing users in the Chinese context. It was important to recognize that the adoption of mobile payments in online taxi services might have differed from other contexts due to cultural, economic, or technological factors.

The subsequent section focuses on a review of the key literature and the development of hypotheses and conceptual frameworks. The third section of this study describes the methodology adopted, and the fourth section describes the data analysis. The following sections present the discussion, implications, and conclusions of this study.

## Literature review

### Theoretical foundation

Davis [17] introduced the TAM to measure user intentions to adopt new technology. The model was developed based on Fishbein and Ajzen’s [18] theory of reasoned action (TRA). Accordingly, TAM consists of perceived usefulness (PU), perceived ease of use (PE), and attitude. Studies have demonstrated that these three elements are salient, valid, and highly consistent predictors of the intention to adopt a particular technology [19]. However, attitude was not considered in the current study given its focus on AD for e-hailing services; in other words, actual use was deemed more appropriate for this study. Thus, this study focused only on PU and PE. Accordingly, PU refers to the extent of one’s perception of how a particular technology helps enhance task performance, whereas PE refers to the extent of one’s perception of how a particular technology helps one perform a specific task with ease [20].

Prior studies that adopted the TAM agreed on its usefulness and appropriateness in measuring technology acceptance behavior at the individual level [21]. Apart from the TAM, there are other theories such as TAM2 by Venkatesh and Davis [22], the unified theory of acceptance and use of technology (UTAUT) model by Venkatesh et al. [23], and TAM3 by Venkatesh and Bala [24]. However, these theories are not as flexible as the TAM, making them less suitable for exploring the adoption of a particular technology system, such as m-payment [25]. The TAM can be extended by incorporating other potential constructs that are salient to the adoption of a particular technology [26]. Thus, the TAM served as the underlying theoretical basis of this study’s conceptual framework to explore the significant factors that influence IN and AD in e-hailing services in China.

### Development of hypotheses

#### Perceived usefulness

Davis [17] defined PU as one’s perception of how a particular technology can be useful in various ways to enhance overall job performance. Through m-payment, e-hailing users can conveniently provide the exact amount of payment via their mobile devices without relying on conventional payment methods, which require more time and effort [27]. In addition, m-payments offer other benefits, such as cashback and loyalty points, which can be used for future e-hailing rides. These benefits have motivated more e-hailing users to adopt m-payment. Numerous studies have explored the relationship between PU and IN in various contexts [27–30]. These studies identified PU as one of the most significant predictors of IN. In contrast to previous studies on the adoption and acceptance of a single technology, this study examined the perceived usefulness of both mobile payment and e-hailing applications for users, allowing us to explore the compatibility and user acceptance of both technologies. Thus, the following hypothesis is proposed:

*H1*: *PU has significant and positive influence on IN*.

#### Perceived ease of use

Shaw and Sergueeva [31] described PE as consumers’ perceptions of how the use of m-payments can be effortless. Consumers can effortlessly make instant payments through a few clicks on the application or by scanning the corresponding merchant’s quick response (QR) code [32]. Shankar and Datta [33] identified several key attributes that contribute to PE in AD, namely effortless participation, instant transfer, and a simple interface. Alhassany and Faisal [34] identified PE as a significant factor that influences AD. Focusing on consumers in China, Sleiman et al. [35] demonstrated the positive and strong influence of PE on IN. Several other studies [29, 36–38] reported similar findings regarding the statistically significant influence of PE on IN. The preference to adopt m-payment over conventional payment methods is attributed to the ease of using m-payment, as transactions using cash or credit cards are more time-consuming and require more effort [39]. Thus, the following hypothesis is proposed for testing in this study:

*H2*: *PE has significant and positive influence on IN*.

#### Social Influence (SI)

SI can be defined as individuals’ perception of how significant others view the use of a particular technology [40]. When it comes to m-payments, consumers often seek the approval and opinions of their family members, friends, or colleagues, which can influence their intention to adopt (IN) [41]. Positive recognition and feedback regarding m-payments within one’s social circle can serve as motivation to use mobile payments for e-hailing services. Previous studies consistently highlight the positive influence of SI on IN [41–44]. Al Mulhem and Almaiah [45] also found strong evidence for the significant and positive influence of SI on IN. Given that e-hailing companies employ advertising and marketing techniques (e.g., discounts, points redemption) through various channels, including social media, and that some e-hailing apps exclusively accept mobile payments, passive adoption of mobile payments by users is encouraged [46, 47]. Therefore, this study focuses on users’ perceptions and feelings regarding the social impact of mobile payments from the perspective of mobile payments themselves, rather than the impact of e-hailing services. Based on this, the study hypothesized the following:

*H3*: *SI has significant and positive influence on IN*.

#### Facilitating Condition (FC)

FC can be defined as individuals’ beliefs regarding how organizational and technical infrastructure can support the use of a particular system [23]. Proper support, timely assistance, detailed information, and adequate resources significantly motivate consumers to adopt a specific technology [48]. In the case of m-payment, users are expected to possess basic knowledge and have access to suitable infrastructure to utilize mobile payment services. Consumers tend to respond favorably to m-payments when they can conveniently utilize the facilities provided by third-party payment service providers [2]. Moreover, the widespread availability of high-speed internet and smartphones has created a facilitating environment, making m-payment highly convenient and motivating more consumers to adopt this payment method [49]. Previous studies have consistently shown a significant and positive relationship between FC and IN [50–54]. Therefore, in this study, the following hypothesis was tested:

*H4*: *FC has significant and positive influence on IN*.

#### Perceived Security (SE)

SE refers to individuals’ perception of the security of adopting m-payment in terms of financial transactions and personal information [55]. The level of security offered by m-payment systems significantly influences the adoption behavior (AD) of users. Johnson et al. [56] identified SE as a key predictor of intention to adopt (IN). Previous studies have consistently demonstrated a significant and positive relationship between SE and IN [29, 55, 57, 58] Shao et al. [3] also highlighted the importance of SE in building consumers’ trust and encouraging the adoption of m-payments. Users often have concerns and reservations regarding the security of mobile technology, whether it pertains to mobile payments or e-hailing services [59]. This study explored users’ perceived security by considering both mobile payment and e-hailing technologies. It aimed to address and mitigate these security concerns by investigating users’ perspectives and ideas. Therefore, the following hypothesis was tested in this study:

*H5*: *SE has significant and positive influence on IN*.

#### Lifestyle Compatibility (CM)

CM can be defined as individuals’ prior experiences and values that directly impact their intention to adopt a specific technology [52]. Given its significance as a core driver of consumer acceptance, CM has been considered an additional component of the TAM [60]. In the case of m-payments, individuals’ intention to adopt is influenced by their prior experiences, values, and behavioral patterns [61]. For example, a technologically savvy consumer is more likely to adopt m-payment compared to someone who perceives technology usage as difficult [62]. Numerous prior studies have demonstrated the positive influence of lifestyle compatibility with m-payments on intention to adopt [49, 63–65]. Leong et al. [66] found that users who are familiar with m-payments have a more favorable perception of intention to adopt. Similarly, Permana and Indrawati [67] proposed that CM, in terms of prior experience, values, and preferences, reduces uncertainty in using m-payments. Nur and Gosal [68] reported similar findings regarding the direct positive influence of CM on intention to adopt. This study examines the compatibility between users’ lifestyles and the combined use of mobile payments and e-hailing services. By exploring the compatibility of these two technologies, as well as their goals and directions for future sustainable development, the study aims to shed light on the potential synergies between mobile payments and e-hailing services. Therefore, the following hypotheses were tested in this study:

*H6*: *CM has significant and positive influence on IN*.

#### Intention to adopt M-Payment

Intention to adopt, as described by Fishbein and Ajzen [18], refers to individuals’ inclination to adopt a specific technology. Studies on digital payments have extensively explored the relationship between intention to use and actual adoption. For instance, Sivathanu [69] found that the intention to use is the most significant predictor of the adoption of digital payments in India. Makanyeza [70] found a positive influence of the intention to use e-wallets on their adoption. Chopdar et al. [71] reported similar results. Numerous studies have demonstrated the significant influence of intention to use on actual adoption by capturing several motivational elements of people [42]. Several studies have reported a strong positive relationship between IN and AD [72–74]. Thus, the following hypothesis is proposed for testing:

*H7*: *IN has significant and positive influence on AD for e-hailing services*.

**Fig 1:**
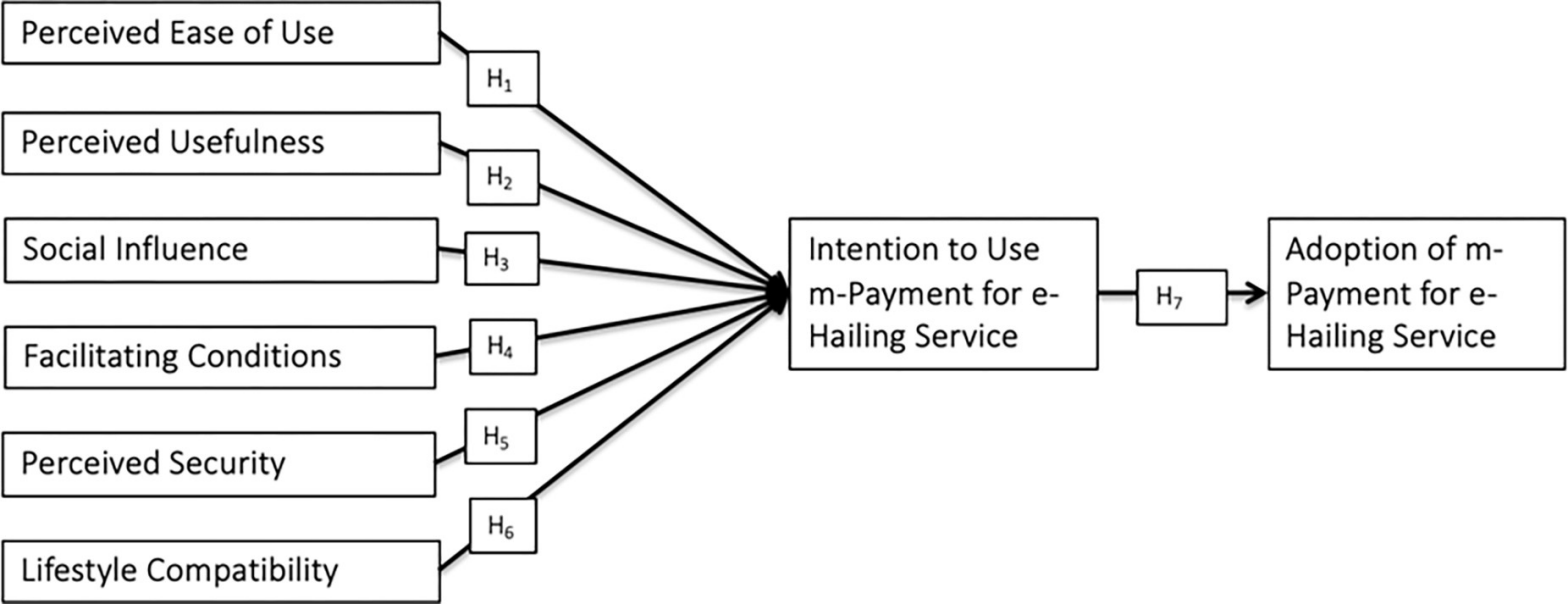
Research framework.

Based on this literature review, we developed a framework for this study (see **Fig 1**).

## Methodology

### Data collection

This cross-sectional study employed a quantitative approach to examine IN and AD among e-hailing users in China. Owing to the COVID-19 restrictions in China at the time of the study, an online survey was conducted. Considering the wide usage of smartphones in the target population, the Chinese version of the questionnaire set link was shared via Chinese online websites and social media platforms such as WeChat and Questionnaire Star. The purpose of the online survey was clearly stated at the top of the online form. As this study falls under business research and management, it does not involve life sciences or medical research on human subjects, and therefore, no formal ethical approval was required (According to the "Ethical Review of Biomedical Research Involving Human Beings," a public source from China’s National Health and Wellness Commission; Link: http://www.gd.gov.cn/zwgk/wjk/zcfgk/content/post_2530813.html). Nonetheless, the study was conducted in accordance with the principles outlined in the Declaration of Helsinki, and written informed consent was obtained from all participating respondents.

The introductory section provided a brief description of the study to help respondents understand the overall purpose and scope. The second section focused on gathering demographic information from the respondents. In the third and final section, respondents provided their responses regarding their intention to adopt and actual adoption of mobile payment for e-hailing services. They were encouraged to share their honest views, opinions, and knowledge about m-payment for e-hailing services. Prior to proceeding, respondents were asked to read and agree to the consent statement at the beginning of the form. Data were collected only from individuals aged 18 years and above. A total of 413 valid questionnaire sets were successfully gathered at the end of the data collection period. The complete dataset for this study can be found in S1. Research Data.

### Instrument

The instrument for this study’s survey was developed using the online platform Questionnaire Star. All respondents were allowed to freely select responses that reflected their opinions and perceptions of the subject [75] on a seven-point Likert scale, with the endpoints of “strongly disagree” (1) and “strongly agree” (7). The wording of certain measurement items was then modified based on the contextual needs and applicability of the study.

All measurement items were adapted from previous studies. The items to measure PU were adapted from Chen et al. [76] and Lwoga and Lwoga [77], whereas the measurement items for PE were adapted from Karjaluoto et al. [78] and Chawla and Joshi [52]. The items used to measure SI and SE were adapted from Humaira and Munazza [79]. The items used to measure FC were adapted from Pandey and Chawla [80]. In addition, the CM measurement items were adapted from Chawla and Joshi [52]. IN was measured using measurement items adapted from Pandey and Chawla [80], whereas AD was measured using measurement items adapted from Karjaluoto et al. [78]. The details of the measurements are presented in S1 Table.

The initial versions of all measurement items were in English. As this study targeted e-hailing users in China, a Chinese version of the instrument was developed to ensure that all respondents could accurately comprehend the content of the online survey. The Chinese experts evaluated and improved the initial draft of the developed instrument to ensure the accuracy and validity of each measurement item.

### Common Method Bias (CMB)

This study addressed the need to minimize any potential bias that may have affected the responses of the respondents. Referring to Kock’s [81] recommendations, a full collinearity test was conducted using IBM SPSS software. The obtained values of the variance inflation factor (VIF) for all constructs are tabulated in Table 1. The VIF values are lower than the threshold value of five, indicating the absence of common method bias from single-source data (CMB) [82].

**Table 1:** Full collinearity test.

Variables	PE	PU	SI	FC	SE	CM	IN	AD
Variance Inflation Factor	1.438	2.294	1.361	1.465	1.438	1.401	2.508	3.763

**Note:** PE: Perceived Ease of Use; PU: Perceived Usefulness; SI: Social Influence; FC: Facilitating Conditions; SE: Perceived Security; CM: Lifestyle Compatibility; IN: Intention to Use m-Payment; AD: Adoption of m-Payment for e-Hailing Service.

**Source:** Author’s data analysis

### Multivariate normality

Direct analysis using methods developed for normal data may invalidate hypothesis testing and lead to unreliable results [1]. Therefore, prior to conducting formal hypothesis testing, many previous studies have recommended using the SmartPLS online tool to measure the Mardia coefficients for multivariate skewness and kurtosis, which are used to assess the multivariate normality hypothesis [61, 83]. The results of Mardia’s test for multivariate normality revealed *p*-values < 0.05, confirming the presence of multivariate non-normality.

### Data analysis

Descriptive analysis was performed using the IBM SPSS software. Considering the presence of multivariate non-normality, this study employed partial least-squares structural equation modelling (PLS-SEM). The results of average variance extraction (AVE), internal consistency reliability, discriminant validity, loadings, and cross-loadings were obtained and evaluated. Testing of hypotheses and multigroup analysis (MGA) were also performed.

## Findings

### Demographic profile of respondents

Referring to Table 2, most of the respondents were female (57.4%) and between 31 and 40 years old (31.0%). Only 9.7% of respondents were over 50 years old. Regarding educational level, the majority of respondents reported having a Bachelor’s degree (48.7%). More than 20.0% of the respondents resided in the northern region of China. In addition, most respondents were self-employed (29.1%) and reported earnings between RMB 1,500 and RMB 3,000 per month (30.0%). Finally, about 39.0% of the respondents had two smartphones, and more than 86% of the total respondents reported using m-payments.

**Table 2:** Demographic profile of respondents.

	n	%		n	%
*Gender*			*Occupation*		
Male	176	42.6	Unemployed	22	5.3
Female	237	57.4	Self-employed	120	29.1
Total	413	100.0	Student	83	20.1
			Housewife	100	24.2
*Age*			Privately employed	88	21.3
20–30 years	123	29.8	Total	413	100.0
31–40 years	128	31.0			
41–50 years	122	29.5	*Monthly Income*		
above 50 years	40	9.7	Less than RMB1500	57	13.8
Total	413	100.0	RMB1500-RMB3000	124	30.0
			RMB3001-RMB4500	84	20.3
*Education*			RM4501-RMB6000	47	11.4
No College Degree	40	9.7	RMB6001-RMB7500	62	15.0
Diploma/Advanced Diploma	110	26.6	More than RMB 7500	39	9.4
Bachelor	201	48.7	Total	413	100.0
Postgraduate Degree	38	9.2			
Others	24	5.8	*Number of Smart Mobile*		
Total	413	100.0	1 Unit	113	27.4
			2 Units	159	38.5
*Location*			3 Units	85	20.6
Northeast China	60	14.5	More than 3 Units	56	13.6
North China	85	20.6	Total	413	100.0
East China	59	14.3			
Central China	46	11.1	*Using mobile payment*		
South China	39	9.4	Yes	356	86.2
Northwest China	58	14	No	57	13.8
Southwest China	40	9.7	Total	413	100.0
Others	26	6.3			
Total	413	100.0			

### Reliability and validity

Several tests were conducted to evaluate the reliability and validity of the constructs. Based on the recommendations of Hair et al. [82], this study measured Cronbach’s alpha, composite reliability, and AVE to evaluate the reliability of the constructs. Referring to Table 3, the Cronbach’s alpha values exceeded the threshold value of 0.7 [82], suggesting the consistency of the constructs. The composite reliability of the constructs was 0.8 (Table 3), confirming their reliability.

**Table 3:** Reliability and validity.

Variables	Items	Mean	Standard Deviation	Cronbach’s Alpha	Dijkstra-Hensele’s rho	Composite Reliability	Average Variance Extracted	Variance Inflation Factor
PE	5	5.119	1.566	0.911	0.913	0.934	0.738	1.386
PU	5	5.085	1.505	0.904	0.909	0.929	0.722	1.436
SI	4	5.072	1.608	0.906	0.906	0.934	0.780	1.322
FC	5	5.126	1.59	0.920	0.927	0.940	0.758	1.443
SE	5	4.48	1.285	0.875	0.907	0.909	0.671	1.372
CM	4	5.09	1.614	0.897	0.901	0.928	0.763	1.391
IN	4	5.154	1.606	0.896	0.897	0.927	0.762	1.000
AD	1	4.06	1.553	1.000	1.000	1.000	1.000	-

**Note:** PE: Perceived Ease of Use; PU: Perceived Usefulness; SI: Social Influence; FC: Facilitating Conditions; SE: Perceived Security; CM: Lifestyle Compatibility; IN: Intention to Use m-Payment; AD: Adoption of m-Payment for e-Hailing Service.

**Source:** Author’s data analysis

Regarding convergent validity, all constructs except SE (0.671) recorded values greater than 0.7. All recorded values were significantly higher than the threshold value of 0.5 [84], confirming the convergent validity of the constructs. The Fornell-Larcker criterion, Heterotrait-Monotrait Ratio (HTMT), and cross-loadings were evaluated to determine the discriminant validity of the constructs. The results are presented in Tables 4 and 5. In particular, the recorded square root values of the AVE exceeded the values of the interconstruct correlation for the corresponding columns and rows, thus satisfying the criterion. Moreover, the recorded HTMT values for the correlations between the constructs did not exceed the threshold value of 0.85 [85]. These results confirmed the discriminant validity of the constructs.

**Table 4:** Discriminant validity.

	PE	PU	SI	FC	SE	CM	IN	AD
Fornell-Larcker Criterion								
PE	0.859							
PU	0.355	0.850						
SI	0.385	0.341	0.883					
FC	0.353	0.434	0.304	0.870				
SE	0.297	0.374	0.356	0.417	0.819			
CM	0.408	0.393	0.316	0.373	0.337	0.873		
IN	0.393	0.341	0.380	0.364	0.368	0.330	0.873	
AD	0.366	0.676	0.383	0.421	0.452	0.374	0.719	1.000
Heterotrait-Monotrait Ratio								
PE								
PU	0.391							
SI	0.424	0.376						
FC	0.385	0.473	0.333					
SE	0.325	0.410	0.390	0.455				
CM	0.452	0.437	0.351	0.410	0.371			
IN	0.433	0.374	0.421	0.398	0.403	0.367		
AD	0.383	0.709	0.402	0.436	0.472	0.395	0.759	

**Note:** PE: Perceived Ease of Use; PU: Perceived Usefulness; SI: Social Influence; FC: Facilitating Conditions; SE: Perceived Security; CM: Lifestyle Compatibility; IN: Intention to Use m-Payment; AD: Adoption of m-Payment for e-Hailing Service.

**Source:** Author’s data analysis

**Table 5:** Loading and cross loadings.

Items	PE	PU	SI	FC	SE	CM	IN	AD
PE1	0.852	0.296	0.321	0.305	0.269	0.307	0.307	0.308
PE2	0.864	0.342	0.322	0.280	0.255	0.365	0.322	0.336
PE3	0.859	0.283	0.357	0.326	0.256	0.377	0.366	0.292
PE4	0.863	0.302	0.334	0.312	0.249	0.405	0.337	0.307
PE5	0.857	0.303	0.318	0.291	0.250	0.296	0.350	0.329
PU1	0.283	0.862	0.277	0.409	0.352	0.345	0.305	0.589
PU2	0.308	0.846	0.289	0.351	0.300	0.324	0.249	0.562
PU3	0.319	0.868	0.306	0.358	0.364	0.349	0.327	0.595
PU4	0.289	0.842	0.267	0.335	0.274	0.318	0.274	0.571
PU5	0.309	0.831	0.307	0.388	0.289	0.331	0.282	0.550
SI1	0.337	0.320	0.875	0.248	0.283	0.308	0.330	0.346
SI2	0.362	0.276	0.888	0.252	0.290	0.261	0.329	0.311
SI3	0.341	0.297	0.881	0.278	0.317	0.258	0.336	0.347
SI4	0.321	0.310	0.888	0.295	0.365	0.288	0.345	0.347
FC1	0.305	0.419	0.238	0.890	0.353	0.347	0.314	0.379
FC2	0.319	0.371	0.272	0.888	0.348	0.333	0.364	0.393
FC3	0.305	0.411	0.283	0.876	0.385	0.349	0.327	0.394
FC4	0.325	0.331	0.264	0.845	0.356	0.279	0.268	0.317
FC5	0.286	0.350	0.267	0.853	0.375	0.309	0.299	0.338
SE1	0.147	0.190	0.168	0.195	0.583	0.156	0.153	0.210
SE2	0.326	0.341	0.340	0.392	0.876	0.320	0.342	0.416
SE3	0.269	0.360	0.352	0.370	0.870	0.279	0.346	0.420
SE4	0.245	0.296	0.300	0.367	0.855	0.276	0.307	0.365
SE5	0.198	0.311	0.258	0.338	0.872	0.314	0.309	0.391
CM1	0.351	0.301	0.286	0.322	0.315	0.880	0.301	0.303
CM2	0.339	0.343	0.220	0.327	0.275	0.879	0.297	0.325
CM3	0.369	0.359	0.295	0.349	0.277	0.862	0.247	0.323
CM4	0.371	0.373	0.305	0.310	0.307	0.871	0.301	0.357
IN1	0.346	0.263	0.321	0.301	0.308	0.280	0.874	0.615
IN2	0.357	0.324	0.360	0.326	0.337	0.274	0.873	0.654
IN3	0.327	0.248	0.325	0.331	0.333	0.339	0.869	0.586
IN4	0.341	0.349	0.317	0.313	0.306	0.263	0.874	0.653
AD1	0.366	0.676	0.383	0.421	0.452	0.374	0.719	1.000

**Note:** PE: Perceived Ease of Use; PU: Perceived Usefulness; SI: Social Influence; FC: Facilitating Conditions; SE: Perceived Security; CM: Lifestyle Compatibility; IN: Intention to Use m-Payment; AD: Adoption of m-Payment for e-Hailing Service.

**Source:** Author’s data analysis

### Testing of hypotheses

As for the testing of the proposed hypotheses, SmartPLS 3.0 was used in this study, specifically the PLS algorithm and bootstrapping technique [82]. Table 6 summarizes the study’s results on the testing of hypotheses.

**Table 6:** Hypothesis testing.

Hypothesis		Beta	Confidence Interval Minimum	Confidence Interval Maximum	*t*- Value	*p*- Value	*r* ^*2*^	*f* ^*2*^	*Q* ^*2*^	Decision
H1	PE -> IN	0.181	0.063	0.282	2.818	0.003		0.033		Accept
H2	PU -> IN	0.081	-0.018	0.178	1.416	0.079		0.006		Reject
H3	SI -> IN	0.168	0.060	0.281	2.553	0.005	0.287	0.030	0.211	Accept
H4	FC -> IN	0.125	0.026	0.234	1.986	0.024		0.015		Accept
H5	SE -> IN	0.146	0.040	0.258	2.190	0.014		0.022		Accept
H6	CM -> IN	0.075	-0.028	0.178	1.254	0.105		0.006		Reject
H7	IN -> AD	0.719	0.660	0.775	20.608	0.000	0.517	1.072	0.514	Accept

**Note:** PE: Perceived Ease of Use; PU: Perceived Usefulness; SI: Social Influence; FC: Facilitating Conditions; SE: Perceived Security; CM: Lifestyle Compatibility; IN: Intention to Use m-Payment; AD: Adoption of m-Payment for e-Hailing Service.

**Source:** Author’s data analysis

The results revealed a statistically insignificant influence of PU (*β* = 0.081, *t* = 1.416, *p* = 0.079) on IN. Thus, H1 was rejected. However, the results demonstrated a statistically significant positive influence of PE (*β* = 0.181, *t* = 2.818, *p* = 0.003) on IN. Thus, H2 was accepted. In other words, the usefulness of m-payment does not influence IN among e-hailing users, but e-hailing users who view the effortlessness of using m-payment favorably are more likely to adopt m-payment for e-hailing services.

Meanwhile, SI (*β* = 0.168, *t* = 2.553, *p* = 0.005), FC (*β* = 0.125, *t* = 1.986, *p* = 0.024) and SE (*β* = 0.146, *t* = 2.190, *p* = 0.014) demonstrated a statistically significant positive influence on IN. Thus, H3, H4, and H5 were supported. In other words, e-hailing users are more likely to adopt m-payment when they receive support from their social circle and are provided with solid infrastructure and security. Surprisingly, the results revealed a statistically insignificant influence of CM (*β* = 0.075, *t* = 1.254, *p* = 0.105) on IN. Thus, H6 was rejected. In other words, e-hailing users do not consider CM when considering AD. The results revealed a statistically significant positive influence of IN (*β* = 0.719, *t* = 20.608, *p* = 0.001) on AD. Therefore, H7 is supported.

Assessment of the structural model involved the values of the path coefficient and *r*^2^. According to Hair et al. [82], *r*^2^ of 0.75 indicates substantial level of predictive accuracy; *r*^2^ of 0.50 indicates moderate level of predictive accuracy; and *r*^2^ of 0.25 indicates weak level of predictive accuracy. As shown in Table 6, *r*^2^ values in this model were 28.7% for IN and 51.7% for AD, indicating moderate predictive relevance.

### Multi-Group Analysis (MGA)

This study employed the measurement invariance of composite model (MICOM) procedure to examine the measurement invariance of two subgroups: female and male respondents. Referring to Table 7, most of the constructs recorded *p*-values greater than 0.05. The path coefficients of the two subgroups were examined using PLS-MGA. Based on the results in Table 7, we found a statistically significant difference in the relationship between IN and AD between male and female respondents (*β* = 0.128, *p* < 0.05). Sex differences between the two subgroups were evident.

**Table 7:** Multi-group analysis.

Associations		Female (N = 237)		Male (N = 176)		Difference		Decision
		Beta	p-value	Beta	p-value	Beta	p-value	
H1	PE->IN	0.039	0.014	-0.157	0.060	-0.011	0.489	No Difference
H2	PU->IN	-0.128	0.469	-0.268	0.048	0.207	0.068	No Difference
H3	SN->IN	0.048	0.009	-0.145	0.198	-0.127	0.176	No Difference
H4	FC->IN	0.028	0.019	0.138	0.147	0.019	0.498	No Difference
H5	CM->IN	-0.061	0.201	-0.190	0.216	0.073	0.365	No Difference
H6	SE->IN	0.007	0.029	-0.331	0.274	-0.071	0.373	No Difference
H7	IN->AD	0.606	0.000	0.701	0.000	0.128	0.035	Difference
Associations		18–40 years (N = 251)		Over 41 years (N = 162)		Difference		Decision
		Beta	p-value	Beta	p-value	Beta	p-value	
H1	PE->IN	0.006	0.045	0.112	0.006	-0.127	0.841	No Difference
H2	PU->IN	0.007	0.038	-0.160	0.397	0.085	0.255	No Difference
H3	SN->IN	0.053	0.011	-0.035	0.099	0.067	0.296	No Difference
H4	FC->IN	0.061	0.004	-0.142	0.455	0.197	0.038	Difference
H5	CM->IN	-0.023	0.112	-0.058	0.181	0.018	0.437	No Difference
H6	SE->IN	-0.068	0.132	0.066	0.017	-0.127	0.836	No Difference
H7	IN->AD	0.645	0.000	0.611	0.000	0.019	0.404	No Difference

**Note:** PE: Perceived Ease of Use; PU: Perceived Usefulness; SI: Social Influence; FC: Facilitating Conditions; SE: Perceived Security; CM: Lifestyle Compatibility; IN: Intention to Use m-Payment; AD: Adoption of m-Payment for e-Hailing Service.

**Source:** Author’s data analysis

Based on these differences, this study employed the MICOM procedure to examine the measurement invariance of the groupings based on respondents’ age: (1) age group of 18–40 years and (2) age group of 41 years or older. Referring to Table 7, this study found that age grouping did not affect most of the hypothesized relationships, except for the relationship between FC and IN. This study found a statistically significant difference in the relationship between FC and IN between the age groups 18–40 years and 41 years or older (*β* = 0.197, *p* < 0.05). The results further revealed a more pronounced influence of FC on IN among respondents aged 18–40 years (*β* = 0.061, *p* < 0.05). In other words, FC is more likely to influence younger users’ intentions to adopt m-payment for e-hailing services.

## Discussion

With respect to the TAM, the current study mainly assessed the factors that influence IN and AD for e-hailing services among Chinese users. An online survey was conducted, and 413 valid questionnaire sets were successfully gathered. Seven hypotheses were proposed for testing. This study obtained sufficient evidence to support five hypotheses (H2, H3, H4, H5, and H7).

First, the results revealed a statistically significant and positive relationship between PE and IN (H2), which supports the results reported by Liébana-Cabanillas et al. [29], Balakrishnan and Shuib [37], and Park et al. [38]. However, PU (H1) and CM (H6) have a statistically insignificant influence on IN. In other words, Chinese e-hailing users do not consider the usefulness or CM of m-payments when it comes to the AD for e-hailing services. Nirmawan and Astiwardhani [86] and Hidayat-ur-Rehman et al. [87] reported similar findings regarding the influence of PU and CM on IN. This situation can be attributed to the extensive proliferation and widespread adoption of mobile payments in China, wherein these payment methods have become deeply ingrained in the daily routines and habits of users and consumers. Consequently, users may overlook the significance of assessing the usefulness and compatibility of mobile payments (CM). This study empirically proved the statistically significant positive influence of SI (H3), FC (H4), and SE (H5) on IN, which supports the findings reported by Chaveesuk et al. [51], Bailey et al. [42], Ivanova and Kim [53]. Put simply, Chinese e-hailing users are more inclined to demonstrate intention to adopt (IN) when they receive support from their social networks and have access to the necessary infrastructure and secure systems for mobile payments. Moreover, perceived security (SE) holds considerable importance for mobile payment users, as they often harbor hesitations and concerns when engaging in various business transactions using mobile payments. These factors subsequently influence their intentions and subsequent behaviors [3]. Additionally, given the emphasis Chinese consumers place on their assets, the security of the payment method when using mobile payments becomes a paramount concern [3, 88]. Lastly, this study provides compelling evidence supporting H7, affirming the substantial impact of IN on actual adoption (AD) in the context of e-hailing services. Hence, it is vital for users to develop a strong intention to adopt.

This study has provided valuable insights into the significant role of users’ perceived ease of use (PE) in influencing the adoption (AD) of e-hailing services through mobile payments. These findings offer valuable guidance for m-payment facilitators in developing effective marketing strategies. By effectively communicating the ease and convenience of using m-payment, particularly for e-hailing services, potential users can be encouraged to embrace this payment method. Therefore, it is crucial for m-payment facilitators to ensure the availability of efficient technical infrastructure and support to enable seamless m-payment experiences for all users. Additionally, the study highlights the importance of leveraging social influence (SI) to promote AD. Third-party m-payment providers can utilize social proof to expand their customer base and encourage the adoption of cashless transactions. Furthermore, given the positive impact of perceived security (SE) on intention to adopt (IN), m-payment facilitators must prioritize robust security features to instill confidence in potential users, encouraging them to transition from conventional payment methods to m-payment and to retain existing users, thus gaining a larger market share.

## Conclusions

This study aimed to assess the influence of PU, PE, SI, FC, SE, and CM on IN and AD for e-hailing services using SEM-MGA. The results demonstrated that PE, SE, SI, FC, and IN are significant factors influencing AD for e-hailing services. However, PU and CM demonstrated a statistically insignificant influence on IN. Through multi-group analysis, this study found that gender also influences the transition from mobile payment intention to actual use, and the degree of influence of facilitating conditions on the intention to use mobile payment varies across age groups. Nonetheless, the incorporation of significant constructs such as SI, SE, and FC into the TAM provided valuable insights into the current literature on m-payment within the context of e-hailing services. Moreover, this study presented valuable insights that can benefit various stakeholders such as m-payment facilitators through their efforts to attract new users and retain existing users.

### Theoretical implications

This study presented several theoretical and practical implications related to AD in e-hailing services. From a theoretical viewpoint, the results and findings of this study contributed to the current literature on m-payments, particularly e-hailing services. Despite extensive studies on AD, the use of AD for e-hailing services has remained underexplored. In addition, AD has been explored in various contexts using constructs similar to TAM. The focus of the current study on the same constructs with respect to the same model (TAM) presented significant insights into the relationships between these constructs in the underexplored context of m-payment for e-hailing services. The impact of e-hailing on TAM theory is significant as it represents a new market that did not previously exist. TAM is a widely used framework for understanding and predicting user acceptance and adoption of new technologies. This theory suggests that perceived usefulness and perceived ease of use are the main factors influencing user behavior. E-hailing services have been successful, in part, because they offer a convenient and affordable alternative to traditional taxi services and are easy to use through mobile applications.

Additionally, TAM was employed as the theoretical basis for the current study. Several new constructs were also considered, extending the existing theoretical framework and structure of the adopted model. Multigroup analysis was also used in this study, and it was discovered that under the classification of groups with different characteristics, such as sex and age, there will be differences in the relationship between some constructs, which can provide guidance for future research. This study provided in-depth and comprehensive insights into IN and AD among e-hailing users in China. This study also empirically demonstrated the importance of considering SI, SE, and lifestyle computability as predictors of IN.

### Practical implications

This study also presented several practical implications for various stakeholders such as third-party facilitators of m-payments and e-hailing providers. First, the findings of this study on IN can substantially benefit third-party payment companies (e.g., Alipay and WeChat Pay) and e-hailing applications (e.g., Didi) that intend to expand their market share and influence in China. Based on the discussed findings, prospective and existing third-party payment companies can make better decisions on the significant determinants considered by potential and existing m-payment users and enhance the quality and delivery of their financial services for e-hailing applications. Thus, the application of m-payments can be significantly improved and expanded to a wider customer base to realize a cashless society in China.

In addition, this study presented empirical evidence of the significant influence of PE and SI on IN. Therefore, it is strategic for third-party payment companies to promote the effortlessness of using m-payment and promote social proof to attract potential users to consider cashless payment methods when using e-hailing services. Moreover, given the significant and positive influence of FC on IN, third-party m-payment companies and e-hailing platforms need cooperation and consistency to promote and deliver adequate technical infrastructure and support for all prospective and existing users; customized facilities or surfaces based on the ages of customers are also worthy of advocacy. At the same time, China’s e-hailing market is experiencing significant growth, with major e-hailing companies such as DDT dominating the market. E-hailing has revolutionized the traditional transport industry by providing a convenient and affordable alternative to traditional taxi and transport services. It has also created new revenue opportunities for groups of drivers whose primary source of income is transport and transportation-type services, creating opportunities to expand businesses that offer their services profitably through apps.

Furthermore, e-hailing and m-payment providers that intend to expand their market share must not overlook the significance of security features, given the significant and positive influence of SE on IN. It is important for users to be educated on the security features of m-payment platforms and the security steps required to use m-payment. It is also necessary for m-payment companies and e-hailing platforms to continuously upgrade their security systems based on current developments in relevant technologies. The mobile payment and e-hailing markets in China are likely to continue to grow in the future, and mobile payments are likely to become more integrated with other services, such as healthcare and education. E-hailing services, such as self-driving cars and drone deliveries, may also expand into new markets. Thus, the results provide detailed insights into the acceptance and adoption intentions of user groups in e-hailing and mobile payment service-based businesses. As these markets continue to evolve, they are likely to continue to contribute to the TAM, providing new insights into user behavior and the adoption of new technologies.

### Limitations and future direction

The current study has several limitations. First, the obtained sample size of 413 respondents in China, which has a population of 1.4 billion, may not be representative of the target population. Therefore, using a larger sample size is recommended. Moreover, the concept of socialism is widely embraced in China. In other words, success is perceived as the success of an entire community or country. Therefore, the findings of this study may not reflect the views of countries that embrace capitalism, such as the United States. Conducting similar studies in capitalist countries may yield contradictory results.

In addition, this study exclusively focused on a single TAM. It is rather complex to assess the full potential of a model for adoption behavior without considering alternatives. Therefore, future research should consider models of technology adoption, such as the motivation model, stimulus-organism-response (S-O-R) model, and the unified theory of acceptance and use of technology (UTAUT) model. Although this study examined the influence of additional constructs, such as SI, SE, CM, and FC, on TAM, other potential key constructs, such as trust, were not considered. Future studies should explore the influence of other potential constructs on e-hailing services.

## Supporting information

S1 Data Research data. (CSV)

S1 Table Survey instrument. (DOCX)

## References

[1] Qu W., Liu H., & Zhang Z. (2019). A method of generating multivariate non-normal random numbers with desired multivariate skewness and kurtosis. *Behavior Research Methods*, 52(3), 939–946. 10.3758/s13428-019-01291-5 31452009

[2] Lee J., Ryu M. H., & Lee D. (2019). A study on the reciprocal relationship between user perception and retailer perception on platform-based mobile payment service. *Journal of Retailing and Consumer Services*, 48, 7–15. 10.1016/j.jretconser.2019.01.007

[3] Shao Z., Zhang L., Li X., & Guo Y. (2019). Antecedents of trust and continuance intention in mobile payment platforms: The moderating effect of gender. *Electronic Commerce Research and Applications*, 33, 100823. 10.1016/j.elerap.2018.100823

[4] Kaur P., Dhir A., Singh N., Sahu G., & Almotairi M. (2020). An innovation resistance theory perspective on mobile payment solutions. *Journal of Retailing and Consumer Services*, 55, 102059. 10.1016/j.jretconser.2020.102059

[5] Pal A., Herath T., De’ R., & Rao H. R. (2020). Contextual facilitators and barriers influencing the continued use of mobile payment services in a developing country: Insights from adopters in India. Information Technology for Development, 26(2), 394–420. 10.1080/02681102.2019.1701969

[6] Ruangkanjanases A., & Techapoolphol C. (2018). Adoption of E-hailing applications: A comparative study between female and male users in Thailand. *Journal of Telecommunication*, *Electronic and Computer Engineering (JTEC)*, 10(1–10), 43–48.

[7] Wu S., Ma E., Wang J., & Li D. (2022). Experience with travel mobile apps and travel intentions—the case of university students in China. *Sustainability*, 14(19*)*, 12603. 10.3390/su141912603

[8] Ye W. M., Chen W., & Fortunati L. (2021). Mobile payment in China: A study from a sociological perspective. *Journal of Communication Inquiry*, 019685992110529. 10.1177/01968599211052965

[9] Kow Y. M., Gui X., & Cheng W. (2017). Special Digital Monies: The design of Alipay and WeChat Wallet for mobile payment practices in China. *Human-Computer Interaction–INTERACT* 2017, 136–155. 10.1007/978-3-319-68059-0_9

[10] Wang X. (2019). Research on development of China E-hailing industry. *SHS Web of Conferences*, 61, 01032. 10.1051/shsconf/20196101032

[11] Ma L., Li T., Wu J., & Yan D. (2018). The impact of E-hailing competition on the Urban Taxi Ecosystem and governance strategy from a rent-seeking perspective: The china E-hailing platform. *Journal of Open Innovation*: *Technology*, *Market*, *and Complexity*, 4(3), 35. 10.3390/joitmc4030035

[12] China Internet Network Information Center (2020). The 46th Statistical Report on the Status of Internet Development in China. Retrieved October 20, 2022, from http://www.gov.cn/xinwen/2020-09/29/content_5548176.htm

[13] Sharif N., & Xing J. L. (2019). Restricted generalizability of city innovation policies: The case of E-hailing in China. *Science and Public Policy*, 46(6), 805–819. 10.1093/scipol/scz031

[14] Yu H., Liu P., Li Z., Zhang G., & Pu Z. (2020). Quantifying significance of young traveler characteristics in travel mode choices impacted by e-hailing services. *Journal of Transportation Engineering*, *Part A*: *Systems*, 146(3). 10.1061/jtepbs.0000310

[15] Shen H., Zou B., Lin J., & Liu P. (2020). Modeling travel mode choice of young people with differentiated E-hailing ride services in Nanjing China. *Transportation research part D*: *transport and environment*, 78, 102216. 10.1016/j.trd.2019.102216

[16] Xing J. L. (2022). Driving as communities: Chinese taxi drivers’ technology, job, and mobility choices under the pressure of e-hailing. *Mobilities*, 1–19.

[17] Davis F.D. (1986) A Technology Acceptance Model for Empirically Testing New End-User Information Systems: Theory and Results. MA: Sloan School of Management, MIT.

[18] Fishbein M., & Ajzen I. (1975). Belief, attitude, intention and behavior: An introduction to theory and research. Reading, MA: Addison-Wesley.

[19] Daragmeh A., Lentner C., & Sági J. (2021). Fintech payments in the era of COVID-19: Factors influencing behavioral intentions of “generation X” in Hungary to use mobile payment. *Journal of Behavioral and Experimental Finance*, 32, 100574. doi: 10.1016/j.jbef.2021.100574 34540592 PMC8442558

[20] Le M. T. H. (2021). Examining factors that boost intention and loyalty to use fintech post-covid-19 lockdown as a new normal behavior. *Heliyon*, 7(8). doi: 10.1016/j.heliyon.2021.e07821 34458639 PMC8379673

[21] Wang J., & Lai J.-Y. (2020). Exploring innovation diffusion of two-sided mobile payment platforms: A System Dynamics Approach. *Technological Forecasting and Social Change*, 157, 120088. 10.1016/j.techfore.2020.120088

[22] Venkatesh V., & Davis F. D. (2000). A theoretical extension of the technology acceptance model: Four longitudinal field studies. *Management Science*, 46, 186–204.

[23] Venkatesh V., Morris M. G., Davis G. B., & Davis F. D. (2003). User acceptance of information technology: toward a unified view. *MIS Quarterly*, 27, 425–478.

[24] Venkatesh V., & Bala H. (2008). Technology acceptance model 3 and a research agenda on interventions. *Decision Sciences*, 39(2), 273–315. 10.1111/j.1540-5915.2008.00192.x

[25] Kim C., Mirusmonov M., & Lee I. (2010). An empirical examination of factors influencing the intention to use mobile payment. *Computers in Human Behavior*, 26(3), 310–322. 10.1016/j.chb.2009.10.013

[26] To A. T., & Trinh T. H. (2021). Understanding behavioral intention to use Mobile Wallets in Vietnam: Extending the TAM model with trust and enjoyment. *Cogent Business & Management*, 8(1). 10.1080/23311975.2021.1891661

[27] Liébana-Cabanillas F., Japutra A., Molinillo S., Singh N., & Sinha N. (2020). Assessment of mobile technology use in the emerging market: Analyzing intention to use M-Payment services in India. *Telecommunications Policy*, 44(9), 102009. 10.1016/j.telpol.2020.102009

[28] Phong N.D., Khoi N.H. and Nhat-Hanh Le A. (2018). Factors affecting mobile shopping: a Vietnamese perspective. *Journal of Asian Business and Economic Studies*, Vol. 25 No. 2, pp. 186–205. 10.1108/JABES-05-2018-0012

[29] Liébana-Cabanillas F., Marinkovic V., Ramos de Luna I., & Kalinic Z. (2018). Predicting the determinants of Mobile Payment Acceptance: A hybrid sem-neural network approach. *Technological Forecasting and Social Change*, 129, 117–130. 10.1016/j.techfore.2017.12.015

[30] Lara-Rubio J., Villarejo-Ramos A. F., & Liébana-Cabanillas F. (2020). Explanatory and predictive model of the adoption of P2P payment systems. *Behaviour & Information Technology*, 40(6), 528–541. 10.1080/0144929x.2019.1706637

[31] Shaw N., & Sergueeva K. (2019). The non-monetary benefits of mobile commerce: Extending UTAUT2 with perceived value. *International Journal of Information Management*, 45, 44–55. 10.1016/j.ijinfomgt.2018.10.024

[32] Mensah I. K. (2021). Predictors of the continued adoption of WECHAT Mobile payment. *Research Anthology on E-Commerce Adoption*, *Models*, *and Applications for Modern Business*, 860–884. 10.4018/978-1-7998-8957-1.ch045

[33] Shankar A., & Datta B. (2018). Factors affecting mobile payment adoption intention: *An Indian perspective*. *Global Business Review*, 19(3_suppl). 10.1177/0972150918757870

[34] Alhassany H., & Faisal F. (2018). Factors influencing the internet banking adoption decision in North Cyprus: An evidence from the partial least square approach of the structural equation modeling. *Financial Innovation*, 4(1). 10.1186/s40854-018-0111-3

[35] Sleiman K. A., Juanli L., Lei H., Liu R., Ouyang Y., & Rong W. (2021). User Trust levels and adoption of mobile payment systems in China: An empirical analysis. *SAGE Open*, 11(4), 215824402110565. 10.1177/21582440211056599

[36] Pipitwanichakarn T. & Wongtada N. (2019). Mobile commerce adoption among the bottom of the pyramid: a case of street vendors in Thailand. *Journal of Science and Technology Policy Management*, Vol. 10 No. 1, pp. 193–213. 10.1108/JSTPM-12-2017-0074

[37] Balakrishnan V., & Shuib N. L. (2021). Drivers and inhibitors for digital payment adoption using the cashless society readiness-adoption model in Malaysia. *Technology in Society*, 65, 101554. 10.1016/j.techsoc.2021.101554

[38] Park J.-S., Ha S., & Jeong S. W. (2020). Consumer acceptance of self-service technologies in Fashion Retail Stores. *Journal of Fashion Marketing and Management*: *An International Journal*, 25(2), 371–388. 10.1108/jfmm-09-2019-0221

[39] Istijanto, & Handoko I. (2022). Customers’ continuance usage of mobile payment during the COVID-19 pandemic. *Spanish Journal of Marketing—ESIC*, 26(3), 345–362. 10.1108/sjme-02-2022-0016

[40] Gani M. O., Rahman M. S., Bag S., & Mia Md. P. (2023). Examining behavioural intention of using Smart Health Care Technology among females: Dynamics of social influence and perceived usefulness. *Benchmarking*: *An International Journal*. 10.1108/bij-09-2022-0585

[41] Migliore G., Wagner R., Cechella F. S., & Liébana-Cabanillas F. (2022). Antecedents to the adoption of mobile payment in China and Italy: An integration of UTAUT2 and innovation resistance theory. *Information Systems Frontiers*. 10.1007/s10796-021-10237-2 PMC8783184 35095331

[42] Bailey A.A., Pentina I., Mishra A.S. & Ben Mimoun M.S. (2017). Mobile payments adoption by US consumers: an extended TAM. *International Journal of Retail & Distribution Management*, *Vol*. 45 *No*. 6, pp. 626–640. 10.1108/IJRDM-08-2016-0144

[43] Alrawad M., Lutfi A., Alyatama S., Elshaer I. A., & Almaiah M. A. (2022). Perception of occupational and environmental risks and hazards among mineworkers: A psychometric paradigm approach. *International Journal of Environmental Research and Public Health*, 19(6*)*, 3371. doi: 10.3390/ijerph19063371 35329057 PMC8955279

[44] Ming K. L., & Jais M. (2022). Factors affecting the intention to use E-wallets during the COVID-19 pandemic. *Gadjah Mada International Journal of Business*, 24(1), 82. 10.22146/gamaijb.64708

[45] Al Mulhem A., & Almaiah M. (2021). A conceptual model to investigate the role of mobile game applications in education during the COVID-19 pandemic. *Electronics*, 10(17), 2106. 10.3390/electronics10172106

[46] Tang Q., & Hu X. (2019). Triggering behavior changes with information and incentives: An active traffic and demand management-oriented review. *Advances in Transport Policy and Planning*, 209–250. 10.1016/bs.atpp.2019.05.002

[47] Osman S., & Din S. A. M. (2022). The Rhapsody of Mobile Applications: A Case Study of Malaysian Travellers. *Handbook of Technology Application in Tourism in Asia*, 681.

[48] Ambarwati R., Harja Y. D., & Thamrin S. (2020). The role of facilitating conditions and user habits: A case of indonesian online learning platform. *The Journal of Asian Finance*, *Economics and Business*, 7(10), 481–489. 10.13106/jafeb.2020.vol7.no10.481

[49] Hussain M., Mollik A.T., Johns R. and Rahman M.S. (2019). M-Payment adoption for bottom of pyramid segment: an empirical investigation. *International Journal of Bank Marketing*, Vol. 37 No. 1, pp. 362–381. 10.1108/IJBM-01-2018-0013

[50] Adel Ali R., & Rafie Mohd Arshad M. (2018). Empirical analysis on factors impacting on intention to use M-learning in basic education in Egypt. *The International Review of Research in Open and Distributed Learning*, 19(2). 10.19173/irrodl.v19i2.3510

[51] Chaveesuk S., Vanitchatchavan P., Wutthirong P., Nakwari P., Jaikua M., & Chaiyasoonthorn W. (2019). The acceptance model toward Cashless Society in Thailand. *Proceedings of the 9th International Conference on Information Communication and Management*. 10.1145/3357419.3357457

[52] Chawla D., & Joshi H. (2021). Degree of awareness and the antecedents of the Digital Media Platform: The Case of Mobile wallets. *FIIB Business Review*, 231971452110234. 10.1177/23197145211023413

[53] Ivanova A., & Kim J. (2021). Acceptance and Use of Mobile Banking in Central Asia: Evidence from Modified UTAUT Model. *Journal of Asian Finance*, *Economics and Business*, 9(2), 0217–0227. 10.13106/jafeb.2022.vol9.no2.0217

[54] Bai B., & Guo Z. (2022). Understanding users’ continuance usage behavior towards digital health information system driven by the Digital Revolution under COVID-19 context: An extended utaut model. *Psychology Research and Behavior Management*, *Volume* 15, 2831–2842. doi: 10.2147/PRBM.S364275 36212806 PMC9532259

[55] Zhang J., Luximon Y., & Song Y. (2019). The role of consumers’ perceived security, perceived control, interface design features, and conscientiousness in continuous use of mobile payment services. *Sustainability*, 11(23), 6843. 10.3390/su11236843

[56] Johnson V. L., Kiser A., Washington R., & Torres R. (2018). Limitations to the rapid adoption of M-payment services: Understanding the impact of privacy risk on M-payment services. *Computers in Human Behavior*, 79, 111–122. 10.1016/j.chb.2017.10.035

[57] Khalilzadeh J., Ozturk A. B., & Bilgihan A. (2017). Security-related factors in extended UTAUT model for NFC based mobile payment in the restaurant industry. *Computers in Human Behavior*, 70, 460–474. 10.1016/j.chb.2017.01.001

[58] Raman P. and Aashish K. (2021). To continue or not to continue: a structural analysis of antecedents of mobile payment systems in India. *International Journal of Bank Marketing*, *Vol*. 39 *No*. 2, pp. 242–271. 10.1108/IJBM-04-2020-0167

[59] Gan Y., Fan H., Jiao W., & Sun M. (2021). Exploring the influence of e-hailing applications on the taxi industry—from the perspective of the drivers. *ISPRS International Journal of Geo-Information*, 10(2), 77. 10.3390/ijgi10020077

[60] Osakwe C. N., Hudik M., Říha D., Stros M., & Ramayah T. (2022). Critical factors characterizing consumers’ intentions to use drones for last-mile delivery: Does delivery risk matter? *Journal of Retailing and Consumer Services*, 65, 102865. 10.1016/j.jretconser.2021.102865

[61] Yang Q., Hayat N., Al Mamun A., Makhbul Z. K., & Zainol N. R. (2022). Sustainable customer retention through social media marketing activities using hybrid SEM-neural network approach. *PLOS ONE*, 17(3), e0264899. doi: 10.1371/journal.pone.0264899 35245323 PMC8896689

[62] Liu P., & Yi S.-ping. (2016). The effects of extend compatibility and use context on NFC Mobile Payment Adoption Intention. *Advances in Human Factors and System Interactions*, 57–68. 10.1007/978-3-319-41956-5_6

[63] Ozturk A.B., Bilgihan A., Salehi-Esfahani S. and Hua N. (2017). Understanding the mobile payment technology acceptance based on valence theory: A case of restaurant transactions. *International Journal of Contemporary Hospitality Management*, Vol. 29 No. 8, pp. 2027–2049. 10.1108/IJCHM-04-2016-0192

[64] Putri A. F., Handayani P. W., & Shihab M. R. (2020). Environment factors affecting individual’s continuance usage of mobile payment technology in Indonesia. *Cogent Engineering*, 7(1), 1846832. 10.1080/23311916.2020.1846832

[65] Agárdi I., & Alt M. A. (2022). Do digital natives use mobile payment differently than digital immigrants? A comparative study between generation X and Z. *Electronic Commerce Research*. 10.1007/s10660-022-09537-9

[66] Leong L.-Y., Hew T.-S., Ooi K.-B., & Wei J. (2020). Predicting mobile wallet resistance: A two-staged structural equation modeling-artificial neural network approach. *International Journal of Information Management*, 51, 102047. 10.1016/j.ijinfomgt.2019.102047

[67] Permana H., & Indrawati. (2020). Addition of lifestyle compatibility and trust in modified UTAUT2 model to analyze continuance intention of customers in using mobile payment. *Managing Learning Organization in Industry 4*.0, 1–7. 10.1201/9781003010814-1

[68] Nur T., & Gosal G. A. (2021). Mobile payment usage in online shopping among gen Z in the Jabodetabek area: Meta-utaut approach. *2021 International Conference on Information Management and Technology (ICIMTech)*. 10.1109/icimtech53080.2021.9535003

[69] Sivathanu B. (2019), Adoption of digital payment systems in the era of demonetization in India: An empirical study, Journal of Science and Technology Policy Management, 10(1), 143–171. 10.1108/JSTPM-07-2017-0033

[70] Makanyeza C. (2017). Determinants of consumers’ intention to adopt mobile banking services in Zimbabwe. *International Journal of Bank Marketing*, 35(6), 997–1017. 10.1108/ijbm-07-2016-0099

[71] Chopdar P. K., Korfiatis N., Sivakumar V. J., & Lytras M. D. (2018). Mobile shopping apps adoption and perceived risks: A cross-country perspective utilizing the unified theory of acceptance and use of Technology. *Computers in Human Behavior*, 86, 109–128. 10.1016/j.chb.2018.04.017

[72] Barry M., & Jan M. T. (2018). Factors influencing the use of m-commerce: An extended technology acceptance model perspective. *International Journal of Economics*, *Management and Accounting*, 26(1), 157–183.

[73] Nizam F., Hwang H. J., & Valaei N. (2018). Measuring the effectiveness of E-wallet in Malaysia. *Big Data*, *Cloud Computing*, *Data Science & Engineering*, 59–69. 10.1007/978-3-319-96803-2_5

[74] Al-Maroof R. A., & Al-Emran M. (2018). Students acceptance of google classroom: An exploratory study using PLS-SEM approach. *International Journal of Emerging Technologies in Learning (IJET)*, 13(06), 112. 10.3991/ijet.v13i06.8275

[75] Mun Y., Khalid H., & Nadarajah D. (2017). Millennials’ Perception on Mobile Payment Services in Malaysia. *Procedia Computer Science*, 124, 397–404.

[76] Chen Y., Li X., & Sun M. (2017). Competitive Mobile Geo Targeting. *Marketing Science*, 36(5), 666–682.

[77] Lwoga E.T. & Lwoga N.B. (2017), User acceptance of mobile payment: the effects of user-centric security, system characteristics and gender, Electronic Journal of *Information Systems in Developing Countries*, 81(3): 1–24.

[78] Karjaluoto H., Shaikh A. A., Leppäniemi M. and Luomala R. (2020), Examining consumers’ usage intention of contactless payment systems, *International Journal of Bank Marketing*, 38(2), 332–35.

[79] Humaira A., & Munazza M. (2018). Study of the Impact of Online Education on Students’ Learning at University Level in Pakistan. *International Journal of Distance Education and E-learning*, 3(2).1–11

[80] Pandey S. and Chawla D. (2018), Online customer experience (OCE) in clothing e-retail: Exploring OCE dimensions and their impact on satisfaction and loyalty–Does gender matter? *International Journal of Retail & Distribution Management*, 46(3), 323–346. 10.1108/IJRDM-01-2017-0005

[81] Kock N. (2015) Common method bias in PLS-SEM: A full collinearity assessment approach. *International Journal of e-Collaboration* 11: 1–10.

[82] Hair J. F., Risher J. J., Sarstedt M., & Ringle C. M. (2019). When to use and how to report the results of PLS-SEM. *European Business Review*, 31(1), 2–24. 10.1108/ebr-11-2018-0203

[83] Al Mamun A., & Fazal S. A. (2018). Effect of entrepreneurial orientation on competency and micro-enterprise performance. *Asia Pacific Journal of Innovation and Entrepreneurship*, 12(3), 379–398. 10.1108/apjie-05-2018-0033

[84] Fornell C., & Larcker D. F. (1981). Structural Equation Models with Unobservable Variables and Measurement Error: Algebra and Statistics. *Journal of Marketing Research*, 18, 382–388.

[85] Henseler J., Ringle C. M., & Sarstedt M. (2014). A new criterion for assessing discriminant validity in variance-based structural equation modeling. *Journal of the Academy of Marketing Science*, 43(1), 115–135. 10.1007/s11747-014-0403-8

[86] Nirmawan H. M., & Astiwardhani W. (2021). The effect of perceived cost, trust, usefulness, and customer value addition on intention to use of go-pay mobile payment services in small traders. *Journal of Business and Management Review*, 2(10), 715–732. 10.47153/jbmr210.2392021

[87] Hidayat-ur-Rehman I., Ahmad A., Akhter F., & Ziaur Rehman M. (2022). Examining consumers’ adoption of Smart wearable payments. *SAGE Open*, 12(3), 215824402211177. 10.1177/21582440221117796

[88] Cao Q., & Niu X. (2019). Integrating context-awareness and UTAUT to explain Alipay user adoption. *International Journal of Industrial Ergonomics*, 69, 9–13. 10.1016/j.ergon.2018.09.004

