# Supplementary material for: Modelling the mass adoption of mobile payment for e-hailing services using SEM-MGA
Source: PLoS One. 2023 Oct 13;18(10):e0287300. doi: 10.1371/journal.pone.0287300 (PMC10575491; doi:10.1371/journal.pone.0287300)
Supplement: S1 Table — (DOCX) [file pone.0287300.s002.docx]

**S1 Table. Survey Instrument**

| Perceived Ease of Use | |
| --- | --- |
| PE1 | Mobile Payment is easy to use for e-hailing service |
| PE2 | Operating mobile payment transaction is easy for e-hailing service |
| PE3 | The features of mobile payment are clear and understandable for e-hailing service |
| PE4 | It is easy to get familiar with mobile payment for e-hailing service |
| PE5 | It is easy to become good at using mobile payment for e-hailing service |
| Perceived Usefulness | |
| PU1 | Mobile Payment helps me to pay more quickly for e-hailing service |
| PU2 | Mobile payment increases online transaction efficiency for e-hailing service |
| PU3 | Mobile payment improves my daily productivity for e-hailing service |
| PU4 | Mobile payment saves me time for e-hailing service |
| PU5 | Mobile payment saves me money for e-hailing service |
| Social Influence | |
| SI1 | People who are important to me (e.g., family members, close friends, and colleagues) recommend me using mobile payment for e-hailing service |
| SI2 | People who are important to me view mobile payment as beneficial for e-hailing service |
| SI3 | People who are important to me think it is a good idea to use mobile payment for e-hailing service |
| SI4 | People who are important to me influence me to use mobile payment for e-hailing service |
| Facilitating Conditions | |
| FC1 | I am given the necessary support and assistance to use mobile payment for e-hailing service |
| FC2 | I have the financial and technological resources required to use mobile payment for e-hailing service |
| FC3 | I have access to the software and hardware required to use mobile payment for e-hailing service |
| FC4 | The mobile payment for e-hailing service are well integrated and provided in a stable service infrastructure |
| FC5 | My service provider/operator facilitates the use of mobile payment for e-hailing service |
| Perceived Security | |
| SE1 | I feel secure using my mobile payment for e-hailing service |
| SE2 | I feel mobile payments are secure when transmitting sensitive information for e-hailing service |
| SE3 | It is completely safe to share personal information during mobile payment for e-hailing service |
| SE4 | I make safe and secure transactions when I use mobile payment for e-hailing service |
| SE5 | I feel fully secured overall when I use mobile payment for e-hailing service |
| Lifestyle Compatibility | |
| CM1 | Using mobile payment for e-hailing service is compatible with all aspects of my lifestyle. |
| CM2 | Using mobile payment for e-hailing service fits into my lifestyle |
| CM3 | Using mobile payment for e-hailing service fits well with the way I like to purchase products and services |
| CM4 | Using mobile payment for e-hailing service is completely compatible with my current situation |
| Intention to Use Mobile Payment | |
| IN1 | I intend to use mobile payment for e-hailing service |
| IN2 | I intend to use mobile payment even in the future for e-hailing service |
| IN3 | I intend to recommend to my friends to use mobile payment for e-hailing service |
| IN4 | I intend to recommend to my family members to use mobile payment for e-hailing service |
| Adoption of wearable payment devices | |
| AD2 | On average, how often have you used mobile payment for e-hailing service per month? |
|  | (Never, 1 to 5 times; 6 to 10 times; 11 to 15 times; More than 15 times) |
